# Salivary Carbonic Anhydrase VI and Gustatory Function Among Adults Assessed After Orthognathic Surgery: A Multicenter Cross-Sectional Study

**DOI:** 10.3390/jcm15218670

**Published:** 2026-11-06

**Authors:** Adel Alenazi

**Affiliations:** Oral and Maxillofacial Surgery, Department of Oral and Maxillofacial Surgery and Diagnostic Sciences, College of Dentistry, Prince Sattam Bin Abdullaziz University, Al-Kharj 16273, Saudi Arabia; a.alenazi@psau.edu.sa

**Keywords:** carbonic anhydrase VI, taste, saliva, orthognathic surgery, biomarkers, salivary glands, taste disorders

## Abstract

**Background/Objectives:** Biological determinants of interindividual variation in gustatory function following orthognathic surgery remain poorly understood. This study investigated the association between salivary carbonic anhydrase VI (CA VI; gustin) concentration and objective whole-mouth gustatory function. **Methods:** This multicenter cross-sectional study included 100 adults following orthognathic surgery at three centers. Whole-mouth taste-recognition thresholds for sweet, salty, sour, and bitter stimuli were categorized from C1 (greatest sensitivity) to C6 (poorest sensitivity). Unstimulated salivary CA VI concentration and flow rate were measured under standardized conditions. Separate proportional-odds ordinal logistic regression models assessed associations between CA VI and each taste threshold, adjusting for age, sex, smoking status, salivary flow rate, postoperative interval, and surgical territory. Associations were expressed as adjusted odds ratios (aORs) per doubling of CA VI, with Holm correction across four primary outcomes. **Results:** Higher CA VI was associated with better thresholds for sweet (aOR 2.18, 95% CI 1.30–3.65; *p* = 0.007), salty (2.89, 1.67–4.99; *p* < 0.001), sour (1.95, 1.14–3.32; *p* = 0.014), and bitter (2.87, 1.66–4.96; *p* < 0.001) stimuli. Estimates remained similar after center adjustment. Linear sensitivity analyses confirmed associations for sweet, salty, and bitter tastes, whereas the association for sour was less robust. The bitter-taste association persisted in a bias-reduced continuation-ratio model (aOR 2.34, 95% CI 1.50–3.66). **Conclusions:** Higher salivary CA VI was associated with better objective whole-mouth taste-recognition sensitivity across four modalities among adults assessed after orthognathic surgery. CA VI may represent a candidate biological correlate of gustatory function; causality and postoperative change cannot be inferred.

## 1. Introduction

Gustation is a fundamental oral sensory function that contributes to food evaluation, preference, and intake [1,2]. Taste perception involves peripheral taste receptor cells and specialized sensory pathways, but occurs within an oral environment substantially influenced by saliva [3,4,5]. Saliva dissolves and transports tastants to receptor sites, while its flow, buffering capacity, ionic composition, and protein constituents can modify tastant availability and taste-receptor interactions. Accordingly, variation in salivary properties may contribute to differences in taste sensitivity and oral chemosensory perception [4,5]. Because gustatory function influences food selection and eating behavior, disturbances in taste may affect dietary intake, nutritional status, and quality of life [1,2,6]. Objective assessment of gustatory function is therefore relevant for characterizing oral sensory performance and its biological determinants.

Orthognathic surgery provides a clinically relevant setting in which gustatory function may vary according to the anatomical structures involved. Le Fort I osteotomy involves manipulation of the maxilla and palatal tissues, whereas bilateral sagittal split osteotomy (BSSO) is performed in close proximity to neural structures involved in lingual sensory function [7,8]. Previous studies have therefore evaluated gustatory function after Le Fort I, BSSO, and bimaxillary surgery using localized and whole-mouth assessments [9,10]. Gent et al. reported site- and stimulus-specific alterations in palatal and lingual taste function after Le Fort I and sagittal split osteotomy [9]. More recently, Malkoc et al. assessed 35 patients before surgery and at 1, 3, and 6 months after Le Fort I, BSSO, or bimaxillary surgery and found generally limited postoperative changes, with selected effects differing by taste modality, procedure, and follow-up time [10]. Thus, existing evidence has primarily examined postoperative temporal changes and procedure-related differences in gustatory measures [9,10], providing limited insight into biological factors underlying interindividual variation in taste function among patients assessed after surgery.

Saliva may provide one such biological link because its composition, in addition to its physical properties, can influence oral chemosensation [5,11]. Several salivary constituents have been investigated in relation to taste, including carbonic anhydrase VI (CA VI), also known as gustin [11]. Gustin was identified as CA VI, a zinc-containing secretory carbonic anhydrase, providing a biological basis for earlier observations linking this protein with taste function [12]. Lower salivary gustin/CA VI concentrations have been reported in individuals with taste disorders and associated taste-bud abnormalities [13], while genetic and experimental evidence has implicated the CA6/gustin system in taste-bud maintenance and taste sensitivity, including bitter-taste perception [14,15]. A small human pilot study also reported a positive association between salivary CA VI concentration and zinc-sulfate taste acuity [16]. Other clinical work has linked changes in salivary CA VI with changes in taste function, although the available evidence remains limited and heterogeneous across populations, salivary measures, and taste-assessment methods [17]. These findings support CA VI as a biologically plausible candidate correlate of gustatory function, but its relationship with objective taste sensitivity has not been specifically examined in adults following orthognathic surgery.

Accordingly, the present multicenter cross-sectional study investigated whether salivary CA VI concentration is associated with objective whole-mouth gustatory function in adults assessed following orthognathic surgery. Gustatory function was evaluated using taste-recognition thresholds for sweet, salty, sour, and bitter stimuli. We hypothesized that higher salivary CA VI concentrations would be associated with better taste-recognition sensitivity after adjustment for demographic, clinical, salivary, postoperative, and surgical factors. As an exploratory objective, we examined whether the CA VI–gustatory association differed according to the anatomical surgical territory involved.

## 2. Materials and Methods

### 2.1. Study Design and Considerations

This multicenter cross-sectional observational study investigated the association between salivary carbonic anhydrase VI (CA VI) concentration and objective gustatory function in adults following orthognathic surgery. Participants underwent a standardized assessment at a predefined postoperative time point, including objective whole-mouth gustatory testing and salivary CA VI measurement. Demographic, clinical, and surgical characteristics were recorded as covariates. The study was designed and reported in accordance with the Strengthening the Reporting of Observational Studies in Epidemiology (STROBE) recommendations for cross-sectional studies. All participating centers operated under the General Organization for Teaching Hospitals and Institutes (GOTHI), Egypt. The study was conducted at Ahmed Maher Teaching Hospital, Mataria Teaching Hospital, and Al-Sahel Teaching Hospital. The study protocol was approved by the Research Ethics Committee of the General Organization for Teaching Hospitals and Institutes (GOTHI), Egypt (approval no. HAM00522). This central ethical approval covered participant recruitment and study procedures across all three participating centers. Written informed consent was obtained from all participants prior to enrollment. The study was conducted in accordance with the principles of the Declaration of Helsinki.

### 2.2. Study Setting and Recruitment

The study was conducted across three participating oral and maxillofacial surgery centers in Egypt, providing specialized orthognathic surgical services. All three centers operated under GOTHI and therefore shared a common governmental organizational and institutional governance framework. Participants were consecutively recruited from the outpatient oral and maxillofacial surgery clinics between January 2024 and March 2025. The predefined eligibility window extended from 0 to 24 months after orthognathic surgery, and each participant underwent a single cross-sectional assessment within this window. The exact interval between surgery and study assessment was recorded in months for each participant and included as a continuous covariate in the multivariable analyses. Potentially eligible patients were identified during routine postoperative follow-up and screened according to the predefined eligibility criteria. Eligible participants were invited to undergo the study assessment within the predefined postoperative assessment window.

A standardized study protocol was implemented across all participating centers. All gustatory assessments at the three participating centers were performed by the same trained investigator; therefore, no between-assessor calibration or inter-assessor reliability assessment was required. The same standardized protocol was used at all centers for participant preparation, taste-solution administration, concentration and modality sequence, timing, rinsing procedures, recognition-threshold determination, and data recording. The investigator was blinded to participants’ surgical information and salivary CA VI concentrations at the time of gustatory assessment. CA VI results were unavailable during taste testing because laboratory measurements were performed after completion of the clinical assessments.

### 2.3. Study Population

The study population comprised adults who had undergone orthognathic surgery for the correction of dentofacial deformities and underwent the standardized postoperative study assessment. Participants were eligible if they were aged ≥18 years, had undergone orthognathic surgery, and were able to complete the gustatory assessment and provide an adequate saliva sample. Participants were excluded if they had a pre-existing gustatory disorder; a neurological condition known to affect taste; active oral or mucosal disease that could influence gustatory function; a recent upper respiratory tract infection or COVID-19; previous head and neck radiotherapy; systemic conditions known to substantially affect gustatory or salivary function, including Sjögren syndrome or other significant salivary-gland disease, uncontrolled diabetes mellitus, or chronic renal disease; or current use of medications with recognized effects on taste or salivary secretion, including anticholinergic, psychotropic, antihypertensive, or antihistamine medications. Relevant medical history and current medication use were assessed during eligibility screening to identify conditions or medications known to substantially affect taste perception or salivary secretion. The type and extent of orthognathic surgery and the interval between surgery and study assessment were recorded for each participant.

**Sample Size Calculation:** Because no directly comparable study provided an effect size suitable for a model-specific sample-size calculation for the planned ordinal outcome analysis, a correlation-based approach was used to establish a pragmatic recruitment target. Assuming a moderate association (r = 0.30), a two-sided α of 0.05, and 80% power, 85 evaluable participants were required based on Fisher’s z transformation. Allowing approximately 15% non-evaluable or incomplete assessments increased the recruitment target to 100 participants. This calculation was not model-specific and therefore did not constitute a formal power calculation for the final multivariable proportional-odds models with six ordered outcome categories and multiple covariates. Accordingly, the precision of the resulting effect estimates was characterized using their 95% confidence intervals. The four primary gustatory outcomes were the whole-mouth taste-recognition thresholds for sweet, salty, sour, and bitter tastes, based on the standardized taste-testing approach used by Malkoc et al. [10]. CA VI was the primary salivary exposure of interest based on previous evidence linking salivary CA VI with objective gustatory measures [18].

### 2.4. Gustatory Function Assessment

Gustatory function was assessed using a whole-mouth taste recognition threshold test following the concentration series described by Malkoc et al. [10]. Four taste modalities were evaluated using sucrose (sweet), sodium chloride (salty), citric acid (sour), and quinine hydrochloride (bitter). For each tastant, five concentrations (C1–C5) were prepared in ½-log increments, with C1 representing the lowest and C5 the highest concentration (Table 1).

Taste solutions were centrally prepared using distilled water under standardized conditions, stored in clean, tightly sealed containers under refrigeration (2–8 °C), and distributed to the participating centers using the same handling protocol. Before testing, the solutions were brought to room temperature (22–24 °C).

Participants refrained from food intake for 2 h before testing. Solutions were administered at room temperature (22–24 °C) in a fixed modality order: sodium chloride (salty), sucrose (sweet), citric acid (sour), and quinine hydrochloride (bitter). Before each taste series, participants rinsed with distilled water. A 1 min interval was maintained between successive concentrations, and the mouth was rinsed with distilled water before proceeding to the next taste series. For each tastant, 1 mL of solution was drawn into a syringe and sprayed circularly throughout the oral cavity. Concentrations were presented in ascending order from C1 to C5. After each administration, participants expectorated the solution and were asked whether they perceived a taste and, if so, to identify its quality. The first concentration at which the administered tastant was correctly identified was recorded as the recognition threshold (C1–C5). A single correct identification was sufficient to establish the threshold; the concentration was not repeated for confirmatory testing. Failure to correctly identify the tastant at C5 was classified as C6.

The ascending concentration sequence and fixed modality order were retained to maintain procedural consistency with the whole-mouth taste-testing approach described by Malkoc et al. [10]. The ascending procedure provides a standardized operational definition of recognition threshold; however, because participants could anticipate progressively stronger stimuli, anticipation or response-related bias cannot be excluded. Concentration order and taste-modality order were not randomized.

### 2.5. Saliva Collection and CA VI Laboratory Analysis

Unstimulated whole saliva was collected between 09:00 and 11:00 h, with participants seated comfortably in an upright position. Participants refrained from eating, drinking, smoking, and performing oral hygiene procedures for at least 2 h before collection. After swallowing once to clear the mouth, participants allowed saliva to accumulate naturally and passively drooled into a sterile graduated polypropylene tube for 5 min. Saliva collection was performed before gustatory testing to avoid potential effects of repeated taste stimulation on salivary flow or composition. The unstimulated salivary flow rate was calculated as the collected volume divided by the 5 min collection period and expressed as mL/min.

Immediately after collection, samples were maintained under cooled conditions during handling and transport from the participating centers to the central laboratory. Samples were centrifuged at 1000× *g* for 15 min at 2–8 °C to remove particulate material, and the supernatant was aliquoted into sterile polypropylene tubes and stored at −80 °C for less than two months before analysis. Aliquoting was used to minimize freeze–thaw exposure, and samples were thawed only for the planned assay.

Salivary carbonic anhydrase VI (CA VI) concentrations were quantified in a single central laboratory using a commercially available human CA VI double-antibody sandwich enzyme-linked immunosorbent assay (ELISA) kit (SED073Hu; Cloud-Clone Corp., Katy, TX, USA), according to the manufacturer’s instructions. The manufacturer specifies an analytical range of 0.78–50 ng/mL and a minimum detectable concentration of <0.29 ng/mL. Saliva samples were analyzed undiluted and in duplicate, and the mean of the duplicate measurements was used for analysis. A 100 µL volume of sample or standard was added per well. A calibration curve was generated from the kit standards on each assay plate, and CA VI concentrations were calculated from the corresponding standard curve. Absorbance was measured at 450 nm. The study-specific intra-assay and inter-assay coefficients of variation were 9% and 10%, respectively.

Samples from the three study centers were processed using the same laboratory protocol and were distributed across assay runs without grouping analyses by study center. Laboratory personnel performing the CA VI assays were blinded to participants’ gustatory test results and surgical information.

### 2.6. Clinical and Surgical Variables

Demographic and clinical variables recorded for each participant included age, sex, smoking status, relevant systemic diseases, current medications, and history of recent upper respiratory tract infection or COVID-19. The interval between surgery and study assessment was recorded in months.

Surgical characteristics were obtained from the operative records and included the surgical procedure performed (Le Fort I osteotomy, bilateral sagittal split osteotomy [BSSO], or bimaxillary surgery), anatomical territory involved (maxillary, mandibular, or both), direction and magnitude of skeletal movement, and any concomitant surgical procedures.

Salivary flow rate was recorded as a physiological covariate because salivary secretion may influence gustatory function and may be associated with salivary CA VI concentration.

### 2.7. Statistical Analysis

Statistical analyses were performed using R version 4.3.3 (R Foundation for Statistical Computing, Vienna, Austria). Continuous variables were summarized as the mean ± SD or median (IQR), as appropriate, and categorical variables as *n* (%). Salivary CA VI concentration and unstimulated salivary flow rate were natural log-transformed. Salivary flow rate was included as a physiologically relevant adjustment variable rather than being assumed to represent an established causal confounder or mediator of the CA VI–taste relationship. Its inclusion was motivated by the potential contribution of salivary flow to dilution-related variation in measured CA VI concentration and by its possible independent relationship with gustatory function. Because adjustment for salivary flow is not equivalent to assessing CA VI output, a sensitivity analysis used CA VI secretion rate (ng/min), calculated as CA VI concentration × unstimulated salivary flow rate; flow rate was not included separately in this model because it is a component of the secretion-rate exposure.

Taste-recognition thresholds (C1–C6; C1 = greatest and C6 = poorest sensitivity) were analyzed separately using proportional-odds ordinal logistic regression (MASS::polr()). Models included ln(CA VI) as the primary exposure and were adjusted for age, sex, smoking status, ln(salivary flow rate), postoperative interval, and surgical territory. Results are reported as adjusted odds ratios (aORs) per doubling of CA VI with 95% CIs; aOR >1 indicates greater odds of a better threshold category. Holm correction was applied across the four primary CA VI associations using stats::*p*.adjust().

Proportional odds were assessed using likelihood-ratio comparisons with partial proportional-odds models; global and predictor-specific tests were not interpreted when boundary solutions or singular information matrices prevented valid inference. For bitter taste, partial proportional-odds models allowing postoperative interval and salivary flow rate to have non-proportional effects, individually and jointly, were attempted but were not estimable because of boundary solutions and singular information matrices (Supplementary Table S5). Bitter-taste robustness was additionally assessed by collapsing sparse categories and fitting Firth bias-reduced continuation-ratio models, including a joint model with transition-specific intercepts and common covariate effects (brglm2). Model convergence, sparse categories, separation, and influential observations were evaluated. Influence was assessed by leave-one-out refitting using the standardized change in the ln(CA VI) coefficient (threshold 2/√n = 0.20), together with changes in aORs and *p*-values.

Study center was not included in the primary models because all gustatory assessments were performed by the same blinded investigator using an identical standardized protocol across centers, and the center was not considered an a priori biological confounder of the CA VI–taste association. Nevertheless, the study center was included as a fixed effect in sensitivity analyses to evaluate the robustness of the estimates to potential site-level heterogeneity. Additional sensitivity analyses (i) replaced CA VI concentration with secretion rate (concentration × flow rate), omitting flow rate as a separate covariate, and (ii) treated C1–C6 as a numerical score using linear regression (stats::lm()) with HC3 robust covariance estimates (sandwich::vcovHC()) and coefficient inference (lmtest::coeftest()). Exploratory CA VI × surgical-territory interactions were assessed by likelihood-ratio tests, and Spearman correlations described unadjusted associations (stats::cor.test()). Population-averaged predicted probabilities at the 25th, 50th, and 75th percentiles of CA VI were calculated over participants’ observed covariate distributions, with 95% CIs obtained from 4000 parametric-bootstrap draws. The R packages used for statistical analyses included MASS (version 7.3.60.0.1), brglm2 (version 1.1.1), sandwich (version 3.1.3), lmtest (version 0.9.40), and VGAM (version 1.1.14)**.** All tests were two-sided, with *p* < 0.05 considered statistically significant.

## 3. Results

### 3.1. Participant Characteristics and Study Flow

**Figure 1:**
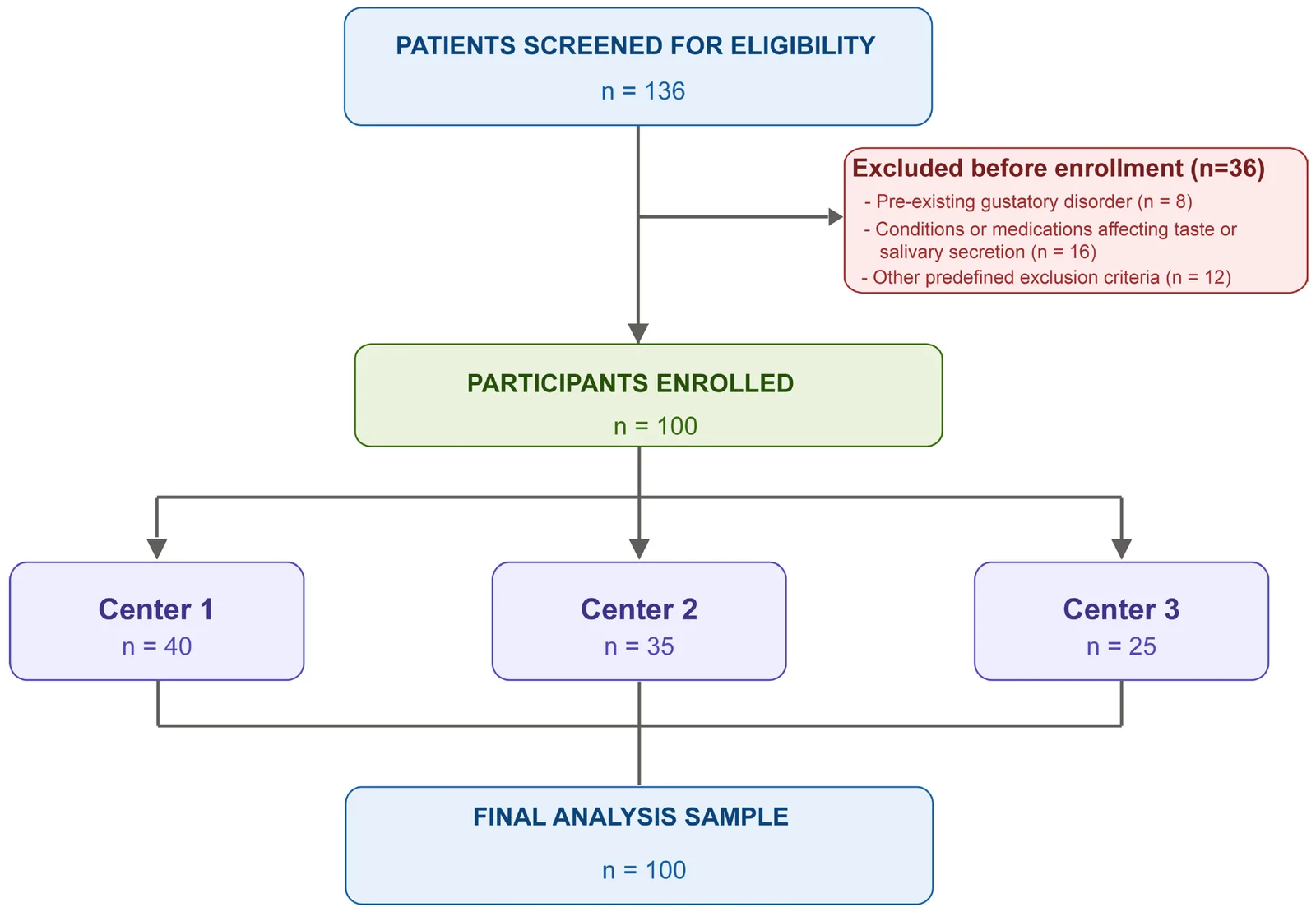
Flow diagram of participant recruitment and inclusion in the analysis.

Of 136 patients screened for eligibility, 36 were excluded before enrollment (pre-existing gustatory disorder, n = 8; conditions or medications affecting taste or salivary secretion, n = 16; other predefined exclusion criteria, n = 12). No patients were excluded because of an inadequate saliva sample or inability to complete the gustatory assessment. The remaining 100 participants constituted the final analytical sample (Figure 1). Participants were drawn from three centers (Center 1, n = 40; Center 2, n = 35; Center 3, n = 25), and all 100 had complete data for salivary CA VI, the four gustatory outcomes, and every covariate. The median postoperative interval was 9.6 months (IQR, 6.5–13.7; range, 4.3–23.9 months), with all participants falling within the predefined 0–24-month eligibility window. Postoperative interval distributions according to study center and surgical procedure are presented in Table 2. Center-specific distributions of salivary CA VI concentration, unstimulated salivary flow rate, and taste-recognition thresholds are provided in Supplementary Table S1.

For each modality, the whole-mouth taste-recognition threshold was recorded in one of six ordered categories, from C1 (greatest sensitivity) to C6 (poorest sensitivity). The modal category was C3 for every modality (sweet 34.0%, salty 32.0%, sour 33.0%, bitter 30.0%). The poorest-sensitivity category (C6) was sparse throughout (sweet, n = 3; salty, n = 3; sour, n = 4; bitter, n = 7), a feature that becomes important for the proportional-odds sensitivity analyses reported below. Bitter thresholds were shifted modestly toward poorer sensitivity relative to the other modalities (C4–C6, 47.0% versus 32–38% for sweet, salty, and sour). The full distributions are presented in Table 2.

### 3.2. Distribution of Taste-Recognition Thresholds and Unadjusted Associations

Salivary CA VI concentration had a median of 6.12 ng/mL (IQR, 4.32–8.70; range, 1.88–20.91 ng/mL) and showed a right-skewed distribution (skewness = 1.39; Shapiro–Wilk *p* < 0.001). Following natural log transformation, the distribution was approximately symmetric (mean ln[CA VI], 1.81 ± 0.51; skewness = −0.01; Shapiro–Wilk *p* = 0.851). Accordingly, ln(CA VI) was used as the exposure in inferential analyses, with effect estimates expressed per doubling of CA VI. Unstimulated whole-saliva flow rate had a median of 0.344 mL/min (IQR, 0.270–0.441; range, 0.112–1.600 mL/min) and was also right-skewed; therefore, it was entered into the regression models on the natural logarithmic scale. Taste-recognition thresholds were distributed across all six ordered categories for each modality (Table 3). C3 was the most frequent category for sweet (34.0%), salty (32.0%), sour (33.0%), and bitter (30.0%) taste. The poorest-sensitivity category, C6, was infrequent, comprising 3.0%, 3.0%, 4.0%, and 7.0% of participants for sweet, salty, sour, and bitter taste, respectively.

**Figure 2:**
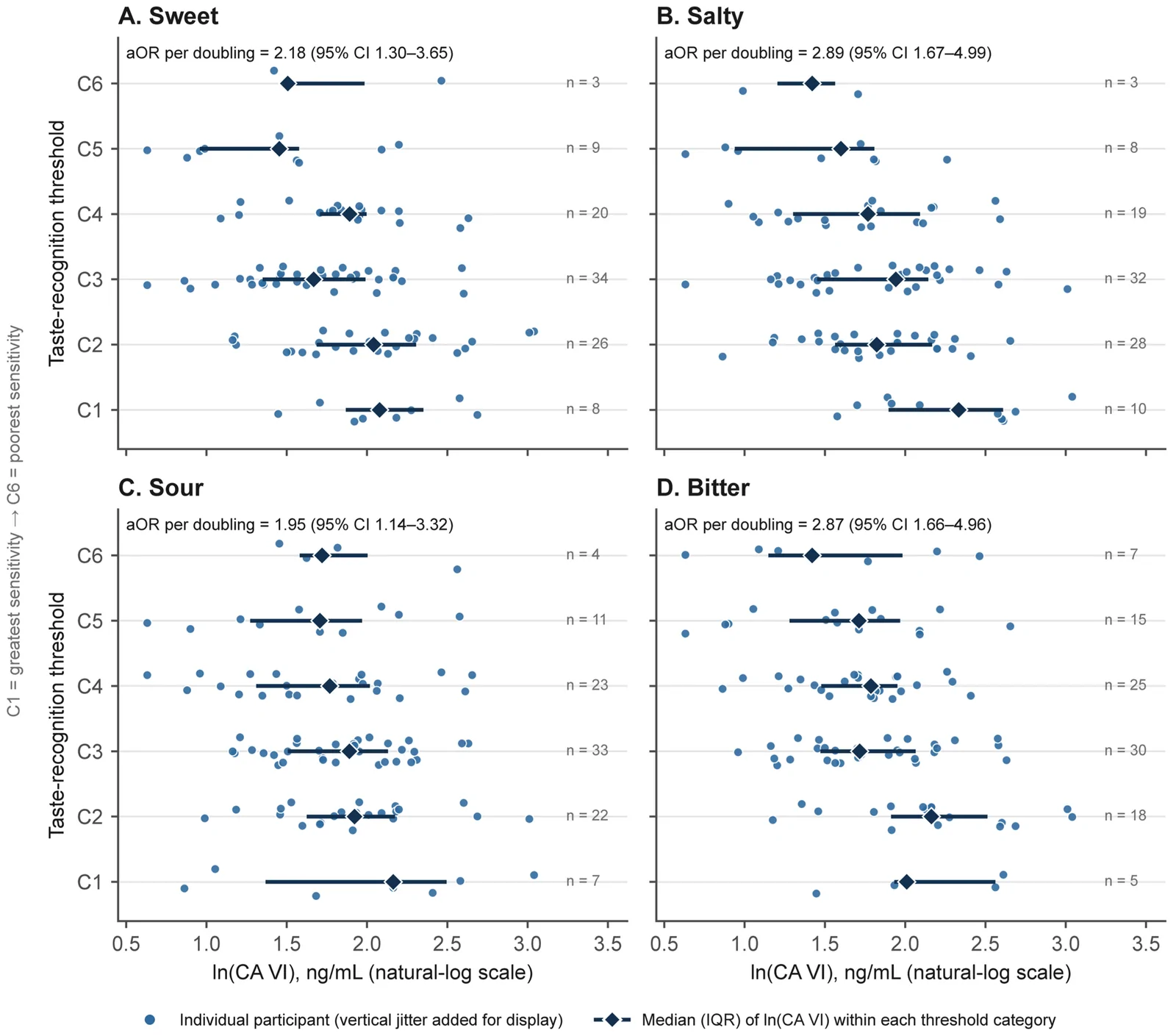
Participant-level associations between salivary CA VI concentration and whole-mouth taste-recognition thresholds for sweet, salty, sour, and bitter taste.

In unadjusted analyses, a higher salivary CA VI concentration was associated with better taste-recognition thresholds for sweet (Spearman ρ = −0.27, *p* = 0.007), salty (ρ = −0.30, *p* = 0.003), sour (ρ = −0.18, *p* = 0.079), and bitter taste (ρ = −0.32, *p* = 0.001); the association reached statistical significance for all tastes except sour, where it was weaker and did not attain significance. Participant-level associations between salivary CA VI concentration and the four taste-recognition thresholds are illustrated in Figure 2.

### 3.3. Primary Associations Between Salivary CA VI and Taste-Recognition Thresholds

**Figure 3:**
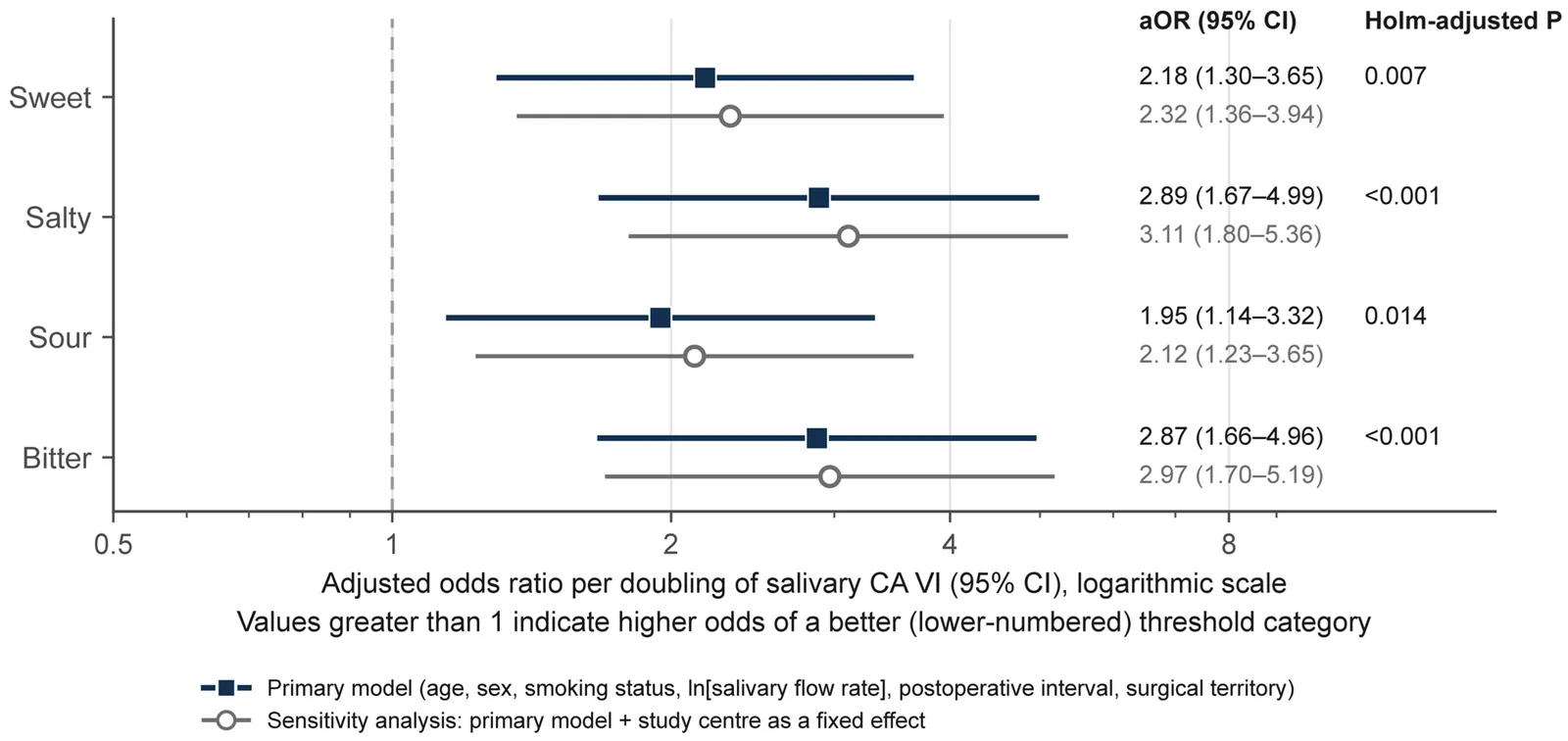
Adjusted odds ratios for a better whole-mouth taste-recognition threshold per doubling of salivary CA VI. Squares show primary proportional-odds models; circles show center-adjusted sensitivity models. Horizontal bars indicate 95% CIs. The dashed line denotes the null (aOR = 1). Models were adjusted for age, sex, smoking status, ln(salivary flow), postoperative interval, and surgical territory. An aOR >1 indicates greater odds of a better (lower-numbered) threshold. Holm-adjusted *p*-values are shown for the primary models.

In the four proportional-odds ordinal logistic regression models—each adjusted for age, sex, smoking status, ln(salivary flow rate), postoperative interval, and surgical territory—a higher salivary CA VI concentration was associated with better (lower-numbered) taste-recognition thresholds for all four modalities; an aOR greater than 1 denotes greater odds of belonging to a better taste-threshold category. Per doubling of CA VI, the adjusted odds of a better threshold were 2.18-fold for sweet (95% CI 1.30–3.65; raw *p* = 0.003, Holm-adjusted *p* = 0.007), 2.89-fold for salty (1.67–4.99; raw *p* < 0.001, Holm-adjusted *p* < 0.001), 1.95-fold for sour (1.14–3.32; raw *p* = 0.014, Holm-adjusted *p* = 0.014), and 2.87-fold for bitter taste (1.66–4.96; raw *p* < 0.001, Holm-adjusted *p* < 0.001), with the strongest associations for salty and bitter taste (Table 4; Figure 3). These associations are associative rather than causal. Complete covariate and cutpoint estimates for the four models are provided in Supplementary Tables S2 and S3, respectively.

### 3.4. Sensitivity Analyses

The principal robustness analyses are consolidated in Table 5, which presents, for each modality, the primary concentration-based model alongside three alternative specifications: a center-adjusted model, a CA VI secretion-rate model, and a numerical (linear) outcome model. The CA VI secretion rate was calculated as CA VI concentration (ng/mL) × unstimulated salivary flow rate (mL/min) = ng/min; because salivary flow is a component of this exposure, it was not separately included as a covariate in the secretion-rate models. Adjustment for study center produced estimates close to the primary models (sweet aOR 2.32, salty 3.11, sour 2.12, bitter 2.97 per doubling). In the secretion-rate models, a higher CA VI secretion rate was associated with better thresholds for sweet (aOR per doubling 1.84, 95% CI 1.27–2.68; Holm-adjusted *p* = 0.003), salty (2.34, 1.59–3.45; Holm-adjusted *p* < 0.001), and bitter taste (2.05, 1.39–3.03; Holm-adjusted *p* < 0.001), whereas the sour association was directionally consistent but not statistically significant (1.37, 0.95–1.96; Holm-adjusted *p* = 0.091). The numerical (linear) outcome model, scoring thresholds from C1 = 1 to C6 = 6 with HC3 robust standard errors, showed the same pattern (sweet β = −0.47 per doubling, Holm-adjusted *p* = 0.008; salty β = −0.61, *p* < 0.001; bitter β = −0.63, *p* = 0.002; sour β = −0.34, *p* = 0.088, not significant). Overall, the sweet, salty, and bitter associations were robust across model specifications and exposure parameterizations, whereas the sour association was the least consistent.

### 3.5. Sequential Analysis of the Sour-Taste Association

To address the apparent discrepancy between the weak unadjusted correlation and the stronger adjusted ordinal association for sour taste, sequential proportional-odds models were examined (Table 6). The CA VI estimate was essentially unchanged across Models 0–2 (aOR per doubling 1.70, 1.71, and 1.72) and strengthened only at Model 3, when postoperative interval and surgical territory were added (1.95, 95% CI 1.14–3.32; *p* = 0.014). In single-covariate analyses, adding postoperative interval alone moved the estimate from 1.70 to 1.91, whereas each covariate changed it by at most 0.03; the strengthening is therefore attributable principally, though cautiously, to adjustment for the postoperative interval. The contrast with the unadjusted correlation is in part a comparison of two different unadjusted analyses, because the Spearman correlation and the log-linear ordinal model do not estimate the same quantity: the unadjusted Spearman correlation was ρ = −0.18 (*p* = 0.079), whereas the unadjusted ordinal model (Model 0) already yielded an aOR of 1.70 (*p* = 0.039).

### 3.6. Clinical Interpretation Using Predicted Probabilities

**Figure 4:**
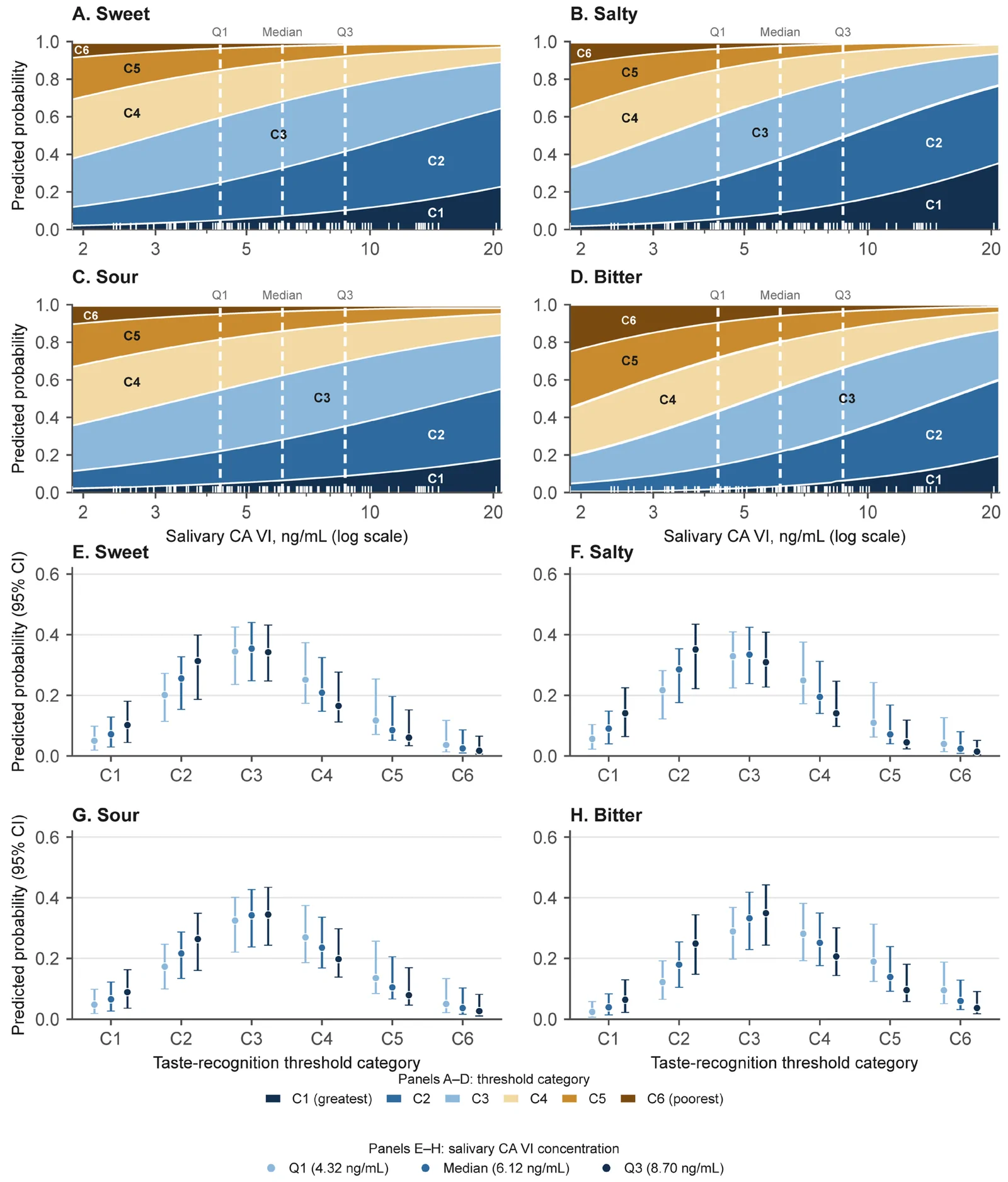
Predicted probabilities of taste-recognition thresholds across salivary CA VI concentrations. (**A**–**D**) Population-averaged predicted probabilities of C1–C6 for sweet, salty, sour, and bitter taste from the adjusted proportional-odds models. Dashed lines indicate Q1 (4.32 ng/mL), the median (6.12 ng/mL), and Q3 (8.70 ng/mL); rug marks indicate observed CA VI values. (**E**–**H**) Predicted probabilities with 95% parametric-bootstrap CIs (4000 draws) at Q1, the median, and Q3. C1 indicates greatest and C6 poorest taste sensitivity. CA VI, carbonic anhydrase VI; CI, confidence interval.

To illustrate the magnitude of the associations, population-averaged predicted probabilities of each threshold category were derived from the four primary models at the 25th percentile (4.32 ng/mL), median (6.12 ng/mL), and 75th percentile (8.70 ng/mL) of the CA VI distribution. Probabilities were obtained by fixing the CA VI at each value while retaining every participant’s observed covariates and averaging across the 100 participants, with 95% bootstrap confidence intervals (Table 7; Figure 4). Across all four modalities, increasing the CA VI shifted the probability distribution toward the better, lower-numbered recognition categories and away from C5–C6. These are population-averaged estimates and do not predict individual patient outcomes.

### 3.7. Model Diagnostics and Exploratory Analyses

Model diagnostics are presented in Supplementary Table S4. Global proportional-odds tests could not be reliably estimated because the fully relaxed models developed complete or quasi-complete separation. Predictor-specific tests were also non-estimable owing to boundary solutions and singular information matrices. CA VI-specific proportional-odds tests were nonsignificant for salty, sour, and bitter taste (*p* = 0.272, 0.837, and 0.702, respectively). For sweet taste, the corresponding test yielded *p* = 0.073; however, boundary estimation limited its interpretation. Because the poorest-sensitivity category was sparse for sweet taste (C6, *n* = 3), a sensitivity analysis combined the adjacent C5 and C6 categories. The resulting CA VI association (aOR per doubling = 2.27; 95% CI 1.34–3.83; *p* = 0.002) was comparable to the primary estimate (aOR = 2.18), supporting robustness to category collapse, although uncertainty regarding proportionality remained.

For bitter taste, combining C5 and C6 yielded an aOR of 2.99 (95% CI 1.71–5.22), compared with 2.87 (95% CI 1.66–4.96) in the primary model. A joint Firth bias-reduced continuation-ratio model incorporating all five transitions also demonstrated a positive CA VI association (aOR per doubling = 2.34; 95% CI 1.50–3.66; *p* < 0.001). There was no statistically significant evidence of variation in the CA VI association across transitions (likelihood-ratio χ^2^ = 2.27; 4 df; *p* = 0.686). These findings supported the robustness of the bitter-taste association under an alternative ordinal modeling framework but did not establish that the proportional-odds assumption was satisfied (Supplementary Table S5).

Finally, potential modification of the CA VI–taste association by surgical territory was examined as an exploratory, hypothesis-generating analysis (Supplementary Table S6). The nominal sour-taste interaction was not interpreted as evidence of a definitive subgroup effect.

## 4. Discussion

This multicenter cross-sectional study found that higher salivary carbonic anhydrase VI (CA VI) concentrations were associated with better objective whole-mouth taste-recognition thresholds for sweet, salty, sour, and bitter tastes after adjustment for demographic, clinical, salivary, postoperative, and surgical factors. The associations were strongest and most consistent for salty and bitter taste and remained materially unchanged after adjustment for study center, with the bitter-taste association further supported by alternative ordinal sensitivity analyses. The sour-taste association warrants more cautious interpretation: although adjustment for postoperative interval strengthened the association in the ordinal model, the finding was attenuated and no longer statistically significant when CA VI concentration was expressed as secretion rate or when taste thresholds were modeled numerically. Thus, the sour association was directionally consistent but less robust across analytical specifications than the associations observed for sweet, salty, and bitter taste. The exploratory territory analysis provided limited evidence of possible modification for sour taste only and should therefore be considered hypothesis-generating.

Higher salivary CA VI concentrations were associated with lower-numbered taste-recognition thresholds, indicating greater objective gustatory sensitivity across all four modalities. This consistent direction is compatible with the role of the salivary environment in gustatory performance, but does not establish CA VI as a causal determinant. CA VI may instead represent a biological correlate of salivary or oral physiological conditions associated with more effective gustatory signaling.

The findings are broadly consistent with previous human evidence linking salivary characteristics with taste function. Walliczek-Dworschak et al. identified differences in salivary composition, including CA VI, between patients with taste disorders and healthy controls and examined these parameters alongside objective chemosensory measures [18]. Zhu et al. similarly reported that changes in gustatory function occurred alongside changes in multiple salivary parameters in a longitudinal pilot study [19]. Although these studies differed from the present investigation in population, design, and taste-assessment methods, they provide convergent evidence that salivary physiology is relevant to gustatory performance. The present findings extend this literature to adults assessed following orthognathic surgery by specifically examining interindividual variation in salivary CA VI and objective whole-mouth taste-recognition thresholds rather than postoperative change itself.

A biological basis for this association is plausible. CA VI, also known as gustin, is a secreted salivary carbonic anhydrase produced predominantly by salivary gland serous acinar cells and involved in local acid–base homeostasis [12,20]. Because tastants interact with taste receptors within the salivary milieu, salivary CA VI could contribute indirectly to the physicochemical environment in which tastant–receptor interactions occur [11,20]. CA VI has also been linked to taste-bud biology, while genetic and experimental evidence has implicated the CA6/gustin system in taste sensitivity, including bitter-taste perception [13,14,15]. However, these mechanisms remain incompletely defined, and CA VI could also reflect broader variation in salivary gland function or oral physiology rather than act as an independent mediator of taste sensitivity. The present findings should therefore be interpreted as biologically plausible associations rather than evidence of a causal pathway.

The strength of the CA VI association varied across taste modalities, with the most consistent associations observed for salty and bitter taste and a less robust association for sour taste in sensitivity analysis. Such heterogeneity is biologically plausible because taste qualities differ in receptor and transduction mechanisms, stimulus chemistry, and interactions with the salivary environment [11,20,21,22]. Saliva influences tastant dissolution, transport, and availability at receptor sites, and these effects may vary according to the physicochemical properties of individual stimuli [5,11]. Differences in peripheral sensitivity and the distribution of recognition thresholds may also affect the precision of modality-specific associations [21,22]. However, the present study was not designed to formally compare CA VI effect estimates between taste modalities; therefore, the apparently stronger salty and bitter associations should be regarded as exploratory rather than as evidence of a modality-specific CA VI effect. Previous clinical studies similarly suggest that relationships between salivary characteristics and gustatory performance may differ across taste modalities [18,19]. Rather, the findings support further investigation of whether the salivary–gustatory relationship varies according to taste modality.

The present findings complement previous longitudinal investigations of gustatory function following orthognathic surgery by addressing a different question. Malkoc et al. assessed 35 patients before and at 1, 3, and 6 months after Le Fort I, BSSO, or bimaxillary surgery and reported generally limited postoperative changes, with selected effects varying by taste modality, procedure, and follow-up time [10]. The 0–24-month eligibility window used in the present study was selected to encompass the clinically relevant continuum of postoperative sensory recovery. Direct gustatory evidence indicates that taste function may continue to evolve during the early postoperative months, with abnormalities remaining detectable in some patients at 6–9 months after orthognathic surgery [9]. Beyond gustation specifically, prospective longitudinal studies have demonstrated that orthognathic surgery-related neurosensory recovery may continue over a longer period, with progressive recovery documented through 24 months following mandibular and maxillary orthognathic procedures [23,24]. These observations provided the biological and clinical rationale for including patients across the first 24 postoperative months, thereby encompassing earlier and later phases of postoperative sensory recovery. In contrast, the present study did not evaluate postoperative change or compare surgical procedures; it examined whether salivary CA VI was associated with interindividual variation in objective whole-mouth taste sensitivity among patients assessed after surgery. This biomarker-based perspective complements procedure- and time-based assessments of gustatory function and remained materially consistent after adjustment for postoperative interval and surgical territory. Thus, the present findings add a salivary biological dimension to the existing orthognathic-surgery literature without implying that CA VI causes postoperative taste changes or that the observed gustatory variation was caused by surgery.

The exploratory interaction analysis provided limited evidence that surgical territory may modify the association between salivary CA VI and gustatory function. No interaction was observed for sweet, salty, or bitter taste, whereas a nominal interaction was detected for sour taste. This finding is uncertain given the small mandibular subgroup (n = 20), wide confidence intervals of territory-specific estimates, and four interaction tests performed without multiplicity correction. Accordingly, the sour interaction does not establish a territory-specific CA VI–taste relationship but provides a hypothesis for prospective studies specifically designed to investigate whether anatomical surgical territory modifies salivary–gustatory relationships.

Clinically, the observed association supports salivary CA VI as a candidate biological correlate for further investigation of gustatory function after orthognathic surgery, rather than as an established diagnostic or predictive biomarker. From a research perspective, these findings support longitudinal studies incorporating preoperative CA VI and objective taste measurements, serial postoperative assessments, standardized salivary-flow measurements, and other validated salivary components. Such designs would help determine whether preoperative or perioperative CA VI levels are associated with subsequent changes in gustatory function and whether CA VI provides information beyond established clinical and salivary determinants.

This study has several strengths. Its multicenter design increases clinical diversity and reduces reliance on a single-center population. Gustatory function was objectively assessed using a standardized whole-mouth taste test across four modalities rather than subjective taste complaints alone. The study also evaluated a biologically plausible salivary factor, incorporated salivary flow as an important physiological covariate, and used adjustment for relevant demographic, clinical, postoperative, and surgical factors. Treating C1–C6 thresholds as ordinal outcomes preserved their inherent ordering, while proportional-odds diagnostics, center-adjusted models, and alternative ordinal sensitivity analyses assessed the robustness of the findings where model estimation was feasible.

Several limitations warrant consideration. The cross-sectional design and absence of preoperative taste measurements preclude establishing temporality, causality, or postoperative change. The broad 0–24-month assessment window may introduce residual time-related heterogeneity despite adjustment for postoperative interval. Sparse C6 observations (3–7 participants across modalities) limited precision and prevented reliable estimation of some proportional-odds diagnostics. Although alternative ordinal models supported the bitter-taste association, they could not establish proportionality. The sour association was less robust across alternative analytical specifications. The exploratory territory interaction was constrained by the small mandibular subgroup, wide confidence intervals, and multiple uncorrected interaction tests and should therefore be considered hypothesis-generating. Although the same trained investigator and standardized protocol were used across all three centers, formal inter-center reliability was not assessed, and residual site-level variation or assessor-related measurement effects cannot be excluded. The gustatory protocol also has measurement limitations: protocol-specific validation and test–retest reliability have not been established; concentrations were presented in ascending order; taste modalities were administered in a fixed order; and a single correct identification defined the recognition threshold without confirmatory repetition. These features may have introduced anticipation, guessing, order, or carryover effects despite standardized rinsing and inter-stimulus intervals.

In addition, CA VI measurement by commercial ELISA is subject to assay-related analytical variability and measurement error despite standardized sample handling and laboratory procedures; the present findings therefore do not establish CA VI as a clinically validated gustatory biomarker. Finally, residual confounding remains possible because oral-health characteristics, nutritional factors such as zinc status and dietary intake, and other salivary characteristics were not comprehensively assessed. Although no patient was excluded because of an inadequate saliva sample or incomplete gustatory assessment, requiring the ability to complete both procedures may have underrepresented individuals with more severe postoperative dysfunction or markedly reduced salivary production, thereby limiting generalizability to more severely affected patients. The direction and magnitude of any resulting selection bias cannot be determined from the present data.

## 5. Conclusions

In this multicenter cross-sectional study of adults assessed following orthognathic surgery, a higher salivary CA VI concentration was associated with better objective whole-mouth taste-recognition thresholds for sweet, salty, sour, and bitter tastes after adjustment for demographic, clinical, salivary, postoperative, and surgical factors. Associations were strongest and most consistent for salty and bitter tastes and remained materially unchanged after adjustment for study center, whereas the association with sour taste was less robust in sensitivity analysis. These findings identify salivary CA VI as a candidate biological correlate of gustatory function following orthognathic surgery but do not establish causality or postoperative change. The exploratory sour taste–territory interaction should be considered hypothesis-generating. Prospective longitudinal studies incorporating preoperative and serial postoperative assessments are needed to characterize within-patient changes in CA VI and gustatory function and to determine their temporal relationship following orthognathic surgery.

## Figures and Tables

**Table 1:** Concentrations used for whole-mouth taste recognition threshold testing.

Taste Modality	Tastant	C1	C2	C3	C4	C5
Sweet	Sucrose (mg/mL)	3.00	9.49	30.00	94.87	300.00
Salty	Sodium chloride (mg/mL)	0.80	2.53	8.00	25.30	80.00
Sour	Citric acid (mg/mL)	0.60	1.90	6.00	18.97	60.00
Bitter	Quinine HCl (mmol/L)	0.010	0.032	0.100	0.316	1.000

C1 represents the lowest concentration and C5 the highest. Concentrations were prepared in ½-log increments according to the concentration series described by Malkoc et al. [10].

**Table 2:** Participant demographic, clinical, surgical, salivary, and postoperative characteristics.

Variable	Overall, N = 100	Postoperative Interval, Median (IQR), Months
Age, years, median (IQR)	28 (25–32)	
Age, years, mean ± SD	28.1 ± 5.2	
Sex, n (%)		
Female	62 (62.0%)	
Male	38 (38.0%)	
Smoking status, n (%)		
Never	61 (61.0%)	
Former	18 (18.0%)	
Current	21 (21.0%)	
Postoperative interval, months, median (IQR)	9.6 (6.5–13.7)	
Surgical procedure, n (%)		
Le Fort I osteotomy	25 (25.0%)	11.3 (6.9–17.0)
BSSO	20 (20.0%)	10.0 (6.3–14.3)
Bimaxillary surgery	55 (55.0%)	7.6 (6.4–12.0)
Surgical territory, n (%)		
Maxillary	25 (25.0%)	
Mandibular	20 (20.0%)	
Both jaws	55 (55.0%)	
Concomitant procedure, n (%)		
Genioplasty	31 (31.0%)	
Other	4 (4.0%)	
None	65 (65.0%)	
Center, n (%)		
Center 1	40 (40.0%)	11.0 (6.6–13.8)
Center 2	35 (35.0%)	7.1 (6.6–13.0)
Center 3	25 (25.0%)	8.4 (5.9–15.5)
Salivary CA VI, ng/mL, median (IQR)	6.12 (4.32–8.70)	
Salivary CA VI, ng/mL, range	1.88–20.91	
ln(CA VI), mean ± SD	1.81 ± 0.51	
Salivary flow rate, mL/min, median (IQR)	0.344 (0.270–0.441)	
Salivary flow rate, mL/min, range	0.112–1.600	

Values are n (%) unless otherwise stated. BSSO, bilateral sagittal split osteotomy; IQR, interquartile range; SD, standard deviation. Age is presented as both median (IQR) and mean ± SD because its distribution departed modestly from normality (Shapiro–Wilk *p* = 0.046). The postoperative interval is the time from surgery to gustatory assessment. Surgical territory corresponds directly to the surgical procedure (Le Fort I, maxillary; BSSO, mandibular; bimaxillary, both jaws). Categories of concomitant procedure are mutually exclusive. Salivary CA VI concentration and unstimulated salivary flow rate were right-skewed and were natural log-transformed for the inferential models. CA VI, carbonic anhydrase VI.

**Table 3:** Distribution of whole-mouth taste-recognition thresholds.

Variable	Overall, N = 100
Sweet taste-recognition threshold, n (%)	
C1	8 (8.0%)
C2	26 (26.0%)
C3	34 (34.0%)
C4	20 (20.0%)
C5	9 (9.0%)
C6	3 (3.0%)
Salty taste-recognition threshold, n (%)	
C1	10 (10.0%)
C2	28 (28.0%)
C3	32 (32.0%)
C4	19 (19.0%)
C5	8 (8.0%)
C6	3 (3.0%)
Sour taste-recognition threshold, n (%)	
C1	7 (7.0%)
C2	22 (22.0%)
C3	33 (33.0%)
C4	23 (23.0%)
C5	11 (11.0%)
C6	4 (4.0%)
Bitter taste-recognition threshold, n (%)	
C1	5 (5.0%)
C2	18 (18.0%)
C3	30 (30.0%)
C4	25 (25.0%)
C5	15 (15.0%)
C6	7 (7.0%)

Taste-recognition thresholds are ordered concentration categories.

**Table 4:** Primary adjusted associations between salivary CA VI concentration and taste-recognition thresholds.

Taste Modality	aOR per Doubling of CA VI (95% CI)	Raw *p*	Holm-Adjusted *p*
Sweet	2.18 (1.30–3.65)	0.003	0.007
Salty	2.89 (1.67–4.99)	<0.001	<0.001
Sour	1.95 (1.14–3.32)	0.014	0.014
Bitter	2.87 (1.66–4.96)	<0.001	<0.001

aORs are from separate proportional-odds ordinal logistic regression models adjusted for age, sex, smoking status, ln(salivary flow rate), postoperative interval, and surgical territory (n = 100 per model). An aOR > 1 indicates greater odds of a better (lower-numbered) threshold category (C1 = greatest sensitivity; C6 = poorest sensitivity) per doubling of salivary CA VI. Raw *p*-values are Wald *p*-values; Holm-adjusted *p*-values account for the four primary CA VI–taste associations. Complete model estimates are provided in Supplementary Table S2. aOR, adjusted odds ratio; CA VI, carbonic anhydrase VI; CI, confidence interval.

**Table 5:** Sensitivity analyses of the association between salivary CA VI and taste-recognition thresholds.

Taste Modality	Primary Concentration Model aOR (95% CI); Holm *p*	Center-Adjusted aOR (95% CI)	Secretion-Rate Model aOR (95% CI); Holm *p*	Linear Model β (95% CI); Holm *p*
Sweet	2.18 (1.30–3.65); 0.007	2.32 (1.36–3.94)	1.84 (1.27–2.68); 0.003	−0.47 (−0.80 to −0.15); 0.008
Salty	2.89 (1.67–4.99); <0.001	3.11 (1.80–5.36)	2.34 (1.59–3.45); <0.001	−0.61 (−0.94 to −0.29); <0.001
Sour	1.95 (1.14–3.32); 0.014	2.12 (1.23–3.65)	1.37 (0.95–1.96); 0.091	−0.34 (−0.74 to 0.05); 0.088
Bitter	2.87 (1.66–4.96); <0.001	2.97 (1.70–5.19)	2.05 (1.39–3.03); <0.001	−0.63 (−1.00 to −0.27); 0.002

Primary models were proportional-odds ordinal logistic regressions adjusted for age, sex, smoking status, ln(salivary flow rate), postoperative interval, and surgical territory; center-adjusted models additionally included study center as a fixed effect. Secretion-rate models used ln(CA VI concentration × unstimulated salivary flow rate) as the exposure and omitted flow rate as a separate covariate. Linear sensitivity models treated thresholds numerically (C1 = 1 to C6 = 6) and used HC3 robust standard errors. Ordinal-model estimates are aORs per doubling of the respective CA VI measure (aOR > 1 indicates better thresholds); linear estimates are β coefficients per doubling of CA VI (β < 0 indicates better thresholds). Holm-adjusted *p*-values were calculated within each set of four CA VI associations. All analyses included 100 participants. aOR, adjusted odds ratio; CA VI, carbonic anhydrase VI; CI, confidence interval.

**Table 6:** Sequential proportional-odds models for the sour taste-recognition threshold.

Model	Terms Included	aOR per Doubling of CA VI (95% CI)	*p*
Model 0	ln(CA VI) only	1.70 (1.03–2.83)	0.039
Model 1	+ age, sex, smoking status	1.71 (1.02–2.84)	0.040
Model 2	+ ln(salivary flow rate)	1.72 (1.03–2.88)	0.039
Model 3	+ postoperative interval, surgical territory (primary model)	1.95 (1.14–3.32)	0.014

Each model is a proportional-odds ordinal logistic regression of the six-level sour taste-recognition threshold (n = 100); terms accumulate down the table, and Model 3 is the primary model. aORs are per doubling of salivary CA VI; an aOR greater than 1 indicates greater odds of a better (lower-numbered) threshold category. *p*-values are raw and not adjusted for multiplicity. aOR, adjusted odds ratio; CA VI, carbonic anhydrase VI; CI, confidence interval.

**Table 7:** Population-averaged predicted probabilities of taste-recognition categories at representative salivary CA VI concentrations.

Taste	CA VI Percentile (ng/mL)	C1	C2	C3	C4	C5	C6
Sweet	25th (4.32)	0.050 (0.019–0.098)	0.201 (0.114–0.272)	0.345 (0.236–0.425)	0.251 (0.173–0.374)	0.117 (0.071–0.253)	0.036 (0.013–0.117)
	50th (6.12)	0.072 (0.030–0.128)	0.256 (0.154–0.327)	0.354 (0.248–0.440)	0.209 (0.147–0.325)	0.085 (0.052–0.196)	0.025 (0.009–0.086)
	75th (8.70)	0.102 (0.044–0.180)	0.313 (0.187–0.399)	0.342 (0.247–0.432)	0.165 (0.112–0.277)	0.061 (0.034–0.152)	0.017 (0.006–0.065)
Salty	25th (4.32)	0.056 (0.022–0.103)	0.217 (0.123–0.281)	0.329 (0.224–0.409)	0.249 (0.172–0.376)	0.109 (0.062–0.242)	0.040 (0.014–0.126)
	50th (6.12)	0.090 (0.040–0.148)	0.286 (0.176–0.354)	0.334 (0.239–0.424)	0.195 (0.140–0.312)	0.071 (0.040–0.168)	0.024 (0.008–0.080)
	75th (8.70)	0.141 (0.064–0.224)	0.351 (0.222–0.435)	0.309 (0.227–0.408)	0.140 (0.097–0.247)	0.045 (0.023–0.118)	0.014 (0.005–0.051)
Sour	25th (4.32)	0.048 (0.018–0.098)	0.173 (0.100–0.247)	0.325 (0.220–0.401)	0.269 (0.186–0.374)	0.136 (0.085–0.257)	0.050 (0.021–0.134)
	50th (6.12)	0.065 (0.026–0.122)	0.217 (0.134–0.287)	0.342 (0.238–0.427)	0.235 (0.168–0.336)	0.105 (0.066–0.206)	0.036 (0.016–0.103)
	75th (8.70)	0.089 (0.036–0.163)	0.264 (0.161–0.349)	0.345 (0.243–0.435)	0.198 (0.138–0.298)	0.079 (0.046–0.169)	0.026 (0.010–0.081)
Bitter	25th (4.32)	0.023 (0.007–0.058)	0.122 (0.065–0.192)	0.289 (0.198–0.368)	0.281 (0.193–0.381)	0.190 (0.124–0.313)	0.095 (0.051–0.188)
	50th (6.12)	0.039 (0.013–0.083)	0.179 (0.104–0.255)	0.332 (0.229–0.418)	0.251 (0.177–0.350)	0.139 (0.091–0.239)	0.060 (0.031–0.129)
	75th (8.70)	0.064 (0.022–0.130)	0.249 (0.148–0.344)	0.349 (0.244–0.443)	0.206 (0.144–0.301)	0.095 (0.058–0.181)	0.036 (0.017–0.090)

Values are the population-averaged predicted probability (95% confidence interval) of each ordered threshold category, obtained from the primary proportional-odds models (adjusted for age, sex, smoking status, ln[salivary flow rate], postoperative interval, and surgical territory) by fixing the CA VI concentration at the 25th, 50th, and 75th percentiles of its observed distribution while holding each participant’s remaining covariates at their observed values and averaging over the 100 participants. C1 denotes the greatest and C6 the poorest taste sensitivity. Within each row, the six point estimates sum to 1.00; confidence intervals are marginal parametric-bootstrap percentile intervals (4000 draws) and are not constrained to sum to 1. CA VI, carbonic anhydrase VI; CI, confidence interval.

## Data Availability

The datasets generated and analyzed during the current study are available from the corresponding author upon reasonable request.

## Supplementary Materials

The following supporting information can be downloaded at https://www.mdpi.com/article/10.3390/jcm15218670/s1, Table S1: Center-specific distributions of salivary CA VI, unstimulated salivary flow rate, and whole-mouth taste-recognition thresholds; Table S2: Complete covariate estimates from the four primary proportional-odds ordinal logistic regression models; Table S3: Cutpoint parameters of the four primary proportional-odds ordinal logistic regression models; Table S4: Diagnostic assessment of the four primary proportional-odds ordinal logistic regression models; Table S5: Bitter-taste sensitivity analyses addressing the proportional-odds assumption; Table S6: Exploratory modification of the CA VI–taste association by surgical territory.
