# Supplementary material for: Salivary Carbonic Anhydrase VI and Gustatory Function Among Adults Assessed After Orthognathic Surgery: A Multicenter Cross-Sectional Study
Source: J Clin Med. 2026 Nov 6;15(21):8670. doi: 10.3390/jcm15218670 (PMC13764246; doi:10.3390/jcm15218670)
Supplement: Supplementary file 1 [file jcm-15-08670-s001.zip › jcm-4584075-supplementary.pdf]

Salivary carbonic anhydrase VI and whole-mouth gustatory function after orthognathic surgery: a multicenter cross-sectional study

Supplementary Tables

Table S1. Center-specific distributions of salivary CA VI, unstimulated salivary flow rate, and whole-mouth taste-recognition thresholds

Variable	Center 1 (n = 40)	Center 2 (n = 35)	Center 3 (n = 25)
CA VI, ng/mL, median (IQR)	6.71 (5.01–7.98)	5.60 (3.82–8.78)	6.08 (4.25–9.06)
Salivary flow rate, mL/min, median (IQR)	0.359 (0.271–0.458)	0.330 (0.265–0.412)	0.366 (0.312–0.428)
Sweet threshold, n (%)			
C1	1 (2.5)	3 (8.6)	4 (16.0)
C2	11 (27.5)	9 (25.7)	6 (24.0)
C3	12 (30.0)	11 (31.4)	11 (44.0)
C4	13 (32.5)	5 (14.3)	2 (8.0)
C5	3 (7.5)	5 (14.3)	1 (4.0)
C6	0 (0.0)	2 (5.7)	1 (4.0)
Salty threshold, n (%)			
C1	3 (7.5)	2 (5.7)	5 (20.0)
C2	10 (25.0)	9 (25.7)	9 (36.0)
C3	15 (37.5)	11 (31.4)	6 (24.0)
C4	8 (20.0)	9 (25.7)	2 (8.0)
C5	2 (5.0)	4 (11.4)	2 (8.0)
C6	2 (5.0)	0 (0.0)	1 (4.0)
Sour threshold, n (%)			
C1	2 (5.0)	4 (11.4)	1 (4.0)
C2	7 (17.5)	5 (14.3)	10 (40.0)
C3	12 (30.0)	12 (34.3)	9 (36.0)
C4	9 (22.5)	11 (31.4)	3 (12.0)
C5	6 (15.0)	3 (8.6)	2 (8.0)
C6	4 (10.0)	0 (0.0)	0 (0.0)
Bitter threshold, n (%)			
C1	2 (5.0)	1 (2.9)	2 (8.0)
C2	5 (12.5)	4 (11.4)	9 (36.0)
C3	16 (40.0)	8 (22.9)	6 (24.0)
C4	12 (30.0)	10 (28.6)	3 (12.0)
C5	5 (12.5)	7 (20.0)	3 (12.0)
C6	0 (0.0)	5 (14.3)	2 (8.0)

Values are median (interquartile range) for continuous variables and n (column %) for taste-recognition threshold categories. C1 denotes the greatest and C6 the poorest taste sensitivity. Percentages are within center and sum to 100 within each taste modality. No formal comparison between centers was performed; center was examined as a fixed effect in the sensitivity analyses reported in the main text. CA VI, carbonic anhydrase VI; IQR, interquartile range.

Table S2. Complete covariate estimates from the four primary proportional-odds ordinal logistic regression models

Taste modality	Predictor (reference category)	β	SE	aOR	95% CI	Raw P
Sweet	ln(CA VI), per 1-unit increase in ln(ng/mL)	1.122	0.381	3.07	1.45–6.48	0.003
	CA VI, per doubling of concentration	0.777	0.264	2.18	1.30–3.65	0.003
	Age, per 1 year	0.008	0.037	1.01	0.94–1.08	0.830
	Sex: male (reference: female)	0.040	0.400	1.04	0.47–2.28	0.921
	Smoking: former (reference: never)	-0.595	0.504	0.55	0.21–1.48	0.238
	Smoking: current (reference: never)	-0.370	0.478	0.69	0.27–1.76	0.440

Taste modality	Predictor (reference category)	β	SE	aOR	95% CI	Raw P
	ln(salivary flow rate), per 1-unit increase in ln(mL/min)	0.569	0.439	1.77	0.75–4.18	0.195
	Postoperative interval, per 1 month	0.050	0.035	1.05	0.98–1.13	0.148
	Surgical territory: mandibular (reference: maxillary)	0.411	0.583	1.51	0.48–4.73	0.480
	Surgical territory: both jaws (reference: maxillary)	0.168	0.474	1.18	0.47–3.00	0.722
Salty	ln(CA VI), per 1-unit increase in ln(ng/mL)	1.530	0.403	4.62	2.10–10.18	<0.001
	CA VI, per doubling of concentration	1.061	0.279	2.89	1.67–4.99	<0.001
	Age, per 1 year	-0.038	0.039	0.96	0.89–1.04	0.331
	Sex: male (reference: female)	-0.246	0.391	0.78	0.36–1.68	0.529
	Smoking: former (reference: never)	0.634	0.505	1.88	0.70–5.07	0.210
	Smoking: current (reference: never)	-0.032	0.488	0.97	0.37–2.52	0.947
	ln(salivary flow rate), per 1-unit increase in ln(mL/min)	0.902	0.415	2.46	1.09–5.55	0.030
	Postoperative interval, per 1 month	0.097	0.037	1.10	1.02–1.19	0.010
	Surgical territory: mandibular (reference: maxillary)	0.355	0.568	1.43	0.47–4.34	0.533
	Surgical territory: both jaws (reference: maxillary)	-0.247	0.458	0.78	0.32–1.92	0.589
Sour	ln(CA VI), per 1-unit increase in ln(ng/mL)	0.961	0.392	2.62	1.21–5.64	0.014
	CA VI, per doubling of concentration	0.666	0.272	1.95	1.14–3.32	0.014
	Age, per 1 year	-0.039	0.035	0.96	0.90–1.03	0.261
	Sex: male (reference: female)	0.196	0.381	1.22	0.58–2.57	0.606
	Smoking: former (reference: never)	0.125	0.494	1.13	0.43–2.98	0.801
	Smoking: current (reference: never)	-0.285	0.495	0.75	0.29–1.98	0.565
	ln(salivary flow rate), per 1-unit increase in ln(mL/min)	-0.113	0.412	0.89	0.40–2.00	0.783
	Postoperative interval, per 1 month	0.055	0.035	1.06	0.99–1.13	0.115
	Surgical territory: mandibular (reference: maxillary)	0.301	0.551	1.35	0.46–3.98	0.585
	Surgical territory: both jaws (reference: maxillary)	0.078	0.473	1.08	0.43–2.73	0.869
Bitter	ln(CA VI), per 1-unit increase in ln(ng/mL)	1.523	0.402	4.58	2.09–10.07	<0.001
	CA VI, per doubling of concentration	1.055	0.278	2.87	1.66–4.96	<0.001
	Age, per 1 year	-0.106	0.040	0.90	0.83–0.97	0.008
	Sex: male (reference: female)	-0.491	0.389	0.61	0.29–1.31	0.207
	Smoking: former (reference: never)	-0.654	0.531	0.52	0.18–1.47	0.218
	Smoking: current (reference: never)	-0.650	0.463	0.52	0.21–1.29	0.160
	ln(salivary flow rate), per 1-unit increase in ln(mL/min)	0.438	0.444	1.55	0.65–3.70	0.325
	Postoperative interval, per 1 month	0.064	0.038	1.07	0.99–1.15	0.094
	Surgical territory: mandibular (reference: maxillary)	-0.235	0.568	0.79	0.26–2.41	0.679
	Surgical territory: both jaws (reference: maxillary)	-0.108	0.465	0.90	0.36–2.23	0.816

Separate proportional-odds (cumulative-logit) models for each taste modality, n = 100 each, all converging without difficulty. The outcome is the six-level taste-recognition threshold ordered from C6 (poorest sensitivity) to C1 (greatest sensitivity), so an aOR above 1 denotes greater odds of a better, lower-numbered category. β and SE are on the log-odds scale and aOR = exp(β); the ln(CA VI) effect is given both per 1-unit increase in ln(CA VI) and per doubling of CA VI, which are the same estimate rescaled and share one P-value. P-values are Wald tests and are unadjusted for multiplicity; only the four primary CA VI associations were Holm-adjusted (main text, Table 3). Model fit: sweet, log-likelihood −148.79, AIC 325.57, McFadden pseudo-R^2 0.048; salty, −143.71, 315.42, 0.087; sour, −155.12, 338.24, 0.027; bitter, −148.70, 325.40, 0.092. Cutpoint parameters are given in Table S3. aOR, adjusted odds ratio; CA VI, carbonic anhydrase VI; CI, confidence interval; SE, standard error.

Table S3. Cutpoint parameters of the four primary proportional-odds ordinal logistic regression models

Taste modality	Cutpoint	Categories separated	θ (log-odds)	SE	95% CI	n at or below cutpoint
Sweet	1	C6 vs C5/C4/C3/C2/C1	-1.576	1.482	-4.481 to 1.329	3
	2	C5 vs C4/C3/C2/C1	0.023	1.408	-2.736 to 2.782	12
	3	C4 vs C3/C2/C1	1.399	1.413	-1.371 to 4.168	32
	4	C3 vs C2/C1	2.950	1.437	0.133 to 5.766	66
	5	C2 vs C1	4.846	1.490	1.926 to 7.766	92
Salty	1	C6 vs C5/C4/C3/C2/C1	-2.229	1.501	-5.170 to 0.713	3
	2	C5 vs C4/C3/C2/C1	-0.738	1.433	-3.546 to 2.070	11
	3	C4 vs C3/C2/C1	0.710	1.431	-2.096 to 3.515	30
	4	C3 vs C2/C1	2.285	1.436	-0.530 to 5.101	62
	5	C2 vs C1	4.274	1.485	1.364 to 7.184	90
Sour	1	C6 vs C5/C4/C3/C2/C1	-1.852	1.420	-4.634 to 0.931	4
	2	C5 vs C4/C3/C2/C1	-0.362	1.364	-3.035 to 2.311	15
	3	C4 vs C3/C2/C1	0.975	1.369	-1.707 to 3.658	38
	4	C3 vs C2/C1	2.465	1.386	-0.251 to 5.182	71
	5	C2 vs C1	4.224	1.441	1.398 to 7.049	93
Bitter	1	C6 vs C5/C4/C3/C2/C1	-3.575	1.506	-6.527 to -0.623	7
	2	C5 vs C4/C3/C2/C1	-2.111	1.455	-4.964 to 0.741	22
	3	C4 vs C3/C2/C1	-0.761	1.438	-3.579 to 2.058	47
	4	C3 vs C2/C1	0.893	1.446	-1.941 to 3.726	77
	5	C2 vs C1	2.915	1.518	-0.059 to 5.889	95

Parameterization: $P(Y \leq j) = \text{logit}^{-1}(\theta_j - x'\beta)$, with the outcome ordered from C6 to C1; cutpoint 1 separates C6 from C5–C1 and cutpoint 5 separates C6–C2 from C1. Cutpoints increase monotonically in every model and their standard errors are of similar magnitude, with none of the inflation that would indicate an unidentified threshold. The final column gives the number of participants at or below each cutpoint and shows where the data are sparse. SE, standard error.

Table S4. Diagnostic assessment of the four primary proportional-odds ordinal logistic regression models

Diagnostic	Sweet	Salty	Sour	Bitter
Panel A. Model adequacy and fit				
Model convergence	Yes	Yes	Yes	Yes
Participants in C1 / C6, n	8 / 3	10 / 3	7 / 4	5 / 7
Largest |β| among covariates	1.12	1.53	0.96	1.52
Largest SE among covariates	0.58	0.57	0.55	0.57
McFadden pseudo-R^2	0.048	0.087	0.027	0.092
Panel B. CA VI association and CA VI-specific proportional-odds assessment				
Wald P for ln(CA VI), primary model	0.003	<0.001	0.014	<0.001
LR test for ln(CA VI), χ^2 (1 df); P	8.85; 0.003	15.08; <0.001	5.97; 0.015	14.57; <0.001
Proportional-odds test for ln(CA VI), LR χ^2 (4 df); P	8.55; 0.073 †	5.15; 0.272	1.44; 0.837	2.18; 0.702
Optimum of the partial model interior to the parameter space	No †	Yes	Yes	Yes
Panel C. Global and predictor-specific proportional-odds assessment				
Global test, all predictors simultaneously (36 df)	Not estimable	Not estimable	Not estimable	Not estimable
Predictor-specific tests for age, sex, smoking status, salivary flow rate, postoperative interval and surgical territory	Not reported ‡	Not reported ‡	Not reported ‡	Not reported ‡

All models included the same covariates and all 100 participants. The proportional-odds assumption for ln(CA VI) was assessed by a likelihood-ratio test comparing the primary model with a partial proportional-odds model in which the ln(CA VI) coefficient varies across the five cumulative cutpoints. † For sweet taste the maximum of the partial model lies on the boundary of the admissible parameter space, where two cumulative probabilities coincide (smallest fitted category probability 6.8×10^{-14}) and the observed-information matrix is singular; the test statistic is reported for completeness but standard asymptotic theory does not strictly apply, and the result is in any case not significant. For salty, sour and bitter taste the optima are interior and the statistics are unchanged from the previous version. ‡ Global tests relaxing all nine coefficients simultaneously require 36 additional parameters in a sample of 100 with sparse extreme categories and were not estimable in any model: every fully relaxed fit developed complete or quasi-complete separation. On validation, the predictor-specific partial proportional-odds fits were likewise found to be boundary solutions with singular information matrices, so no standard errors or valid likelihood-ratio statistics can be obtained from them; they are therefore no longer reported. The two nominal findings previously listed for the bitter model (postoperative interval, P = 0.036; salivary flow rate, P = 0.042) recompute to P = 0.102 and P = 0.055 under the corrected likelihood and are superseded. LR, likelihood ratio; SE, standard error.

Table S5. Bitter-taste sensitivity analyses addressing the proportional-odds assumption

Analysis	aOR per doubling of CA VI	95% CI	P	Comment
Panel A. Partial proportional-odds models (not estimable; not reported as results)				
Model A: postoperative interval non-proportional	Not estimable	—	—	Boundary solution; information matrix singular and not positive definite
Model B: salivary flow rate non-proportional	Not estimable	—	—	Boundary solution; information matrix singular and not positive definite
Model C: both non-proportional	Not estimable	—	—	Boundary solution; information matrix singular and not positive definite
Panel B. Adjacent-category collapse				
Primary model, six categories	2.87	1.66–4.96	<0.001	Reference specification
Five categories, C5 and C6 combined (n = 22 in the merged category)	2.99	1.71–5.22	<0.001	Converged normally
Panel C. Continuation-ratio (sequential) models, transition-specific				
Transition 1: C5–C1 vs C6 — maximum likelihood	2.98	0.70–12.65		n at risk 100, events 93
Transition 1: C5–C1 vs C6 — Firth penalized likelihood	2.15	0.70–6.60		
Transition 2: C4–C1 vs C5 — maximum likelihood	2.64	0.99–7.06		n at risk 93, events 78; separation in the smoking term under ML, resolved by Firth
Transition 2: C4–C1 vs C5 — Firth penalized likelihood	2.29	0.94–5.61		
Transition 3: C3–C1 vs C4 — maximum likelihood	3.00	0.95–9.50		n at risk 78, events 53
Transition 3: C3–C1 vs C4 — Firth penalized likelihood	2.37	0.85–6.62		
Transition 4: C2–C1 vs C3 — maximum likelihood	5.02	1.53–16.49		n at risk 53, events 23
Transition 4: C2–C1 vs C3 — Firth penalized likelihood	3.49	1.21–10.11		
Transition 5: C1 vs C2 — maximum likelihood	Not estimable	—		n at risk 23, events 5; ML did not converge (complete separation), Firth estimable
Transition 5: C1 vs C2 — Firth penalized likelihood	2.09	0.34–12.70		
Panel D. Continuation-ratio models, pooled across transitions				
Joint model, common CA VI effect (maximum likelihood)	2.46	1.56–3.89	<0.001	Transition-specific intercepts, common covariate effects
Joint model, common CA VI effect (Firth)	2.34	1.50–3.66	<0.001	Preferred pooled estimate
Likelihood-ratio test for a CA VI effect varying across transitions	$\chi^2 = 2.27$	4 df	0.686	No evidence of variation across the scale

All analyses use the same 100 participants, the same covariates (age, sex, smoking status, ln[salivary flow rate], postoperative interval, surgical territory) and the same transformations as the primary model, and in every specification an aOR above 1 denotes better (lower-numbered) bitter taste-recognition sensitivity. Panel A: partial proportional-odds models relaxing proportionality for the named covariates could not be estimated; the maximum-likelihood estimates lay on the boundary of the admissible parameter space and the information matrices were singular and not positive definite, so no estimates or intervals are available. The same models could not be fitted by VGAM. Panel C: each continuation-ratio transition models the probability of a better category than the stated reference, among participants who have reached that point on the scale; Firth penalized likelihood was used because two transitions exhibit separation under ordinary maximum likelihood. Panel D: because the continuation-ratio likelihood factorises into independent binomials, a single model with transition-specific intercepts and a common CA VI effect is the appropriate pooled estimator and is identified even where individual transitions are not; it is preferred to inverse-variance pooling of the transition-specific estimates. The continuation-ratio analysis is an exploratory robustness assessment and not a formal test of the proportional-odds assumption, since the continuation-ratio and cumulative-logit models belong to different link families. aOR, adjusted odds ratio; CA VI, carbonic anhydrase VI; CI, confidence interval.

Table S6. Exploratory modification of the CA VI–taste association by surgical territory

Taste modality	Interaction LR χ^2 (2 df)	P	Maxillary (n = 25): aOR (95% CI)	Mandibular (n = 20): aOR (95% CI)	Both jaws (n = 55): aOR (95% CI)
Sweet	0.30	0.863	2.54 (0.91–7.05)	1.59 (0.42–5.99)	2.22 (1.16–4.25)
Salty	2.27	0.322	3.81 (1.39–10.39)	1.05 (0.26–4.17)	3.21 (1.62–6.36)
Sour	6.94	0.031	4.10 (1.55–10.86)	0.42 (0.11–1.66)	2.05 (1.04–4.05)
Bitter	1.27	0.530	2.02 (0.76–5.40)	2.18 (0.58–8.15)	3.73 (1.83–7.60)

Each model added a ln(CA VI) × surgical-territory interaction to the primary model (adjusted for age, sex, smoking status, ln[salivary flow rate] and postoperative interval); n = 100. Territory-specific aORs are per doubling of CA VI within each territory, derived from linear combinations of the main-effect and interaction coefficients with delta-method standard errors. Interaction P-values are likelihood-ratio tests and were not adjusted for multiplicity; the analysis is exploratory and hypothesis-generating. The nominal sour-taste interaction (P = 0.031) is not interpreted as a definitive subgroup effect, and the mandibular subgroup was small (n = 20). All values were reproduced in R and are unchanged from the previous version. aOR, adjusted odds ratio; CA VI, carbonic anhydrase VI; CI, confidence interval; LR, likelihood ratio.
